# The *IJO* Journey: Learn, Educate and Evolve

**DOI:** 10.4103/0301-4738.74016

**Published:** 2011

**Authors:** Ranjit H Maniar, Barun K Nayak

**Affiliations:** Department of Ophthalmology, Shushrusha Hospital, Mumbai, India. E-mail: editor@ijo.in; 1 P. D. Hinduja National Hospital, Veer Savarkar Marg, Mumbai - 400 016, India

Rome was not built in a day and neither is the *Indian Journal of Ophthalmology (IJO)*. Contemporary paradigms are rooted in the past, as will future trends evolve from today’s mores. The strong foundation laid by all preceding editors made the present team’s task just a bit easier in lifting the *IJO* to a truly global stature.

Soon after it assumed office, the new editorial team conceived of an image makeover for the *IJO*. This metamorphosis was aptly depicted in the new cover layout. We retained a dash of Blue to symbolize continuity. The Vertical bar signified our steadfastness, while the dominant White was a sign of the pristine purity of our mindset toward our goals – promoting a scientific temper within the All India Opthalmological Society (AIOS) fraternity.

We also designed a new logo [Fig. 1] to embody our new goals:

**Figure 1:**
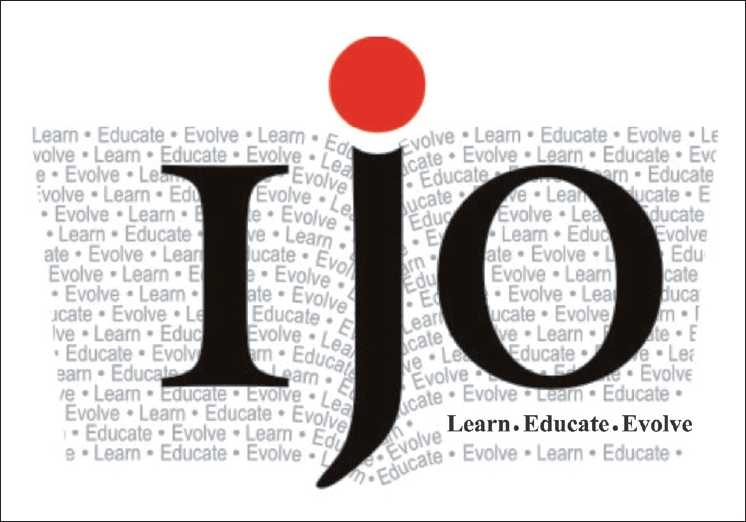
IJO logo

## Learn, Educate and Evolve

We positioned the letters **I j O** over this matrix, with the **j** representing the central binding rib of our mission. The Red Bindi atop denotes the blessings of Ma Saraswati toward our noble endeavor.

As we look back, we feel this is the opportune time to review the progressive advancement of the *IJO* journey over the last 6 years that the present editorial team has been working.

In the first editorial[1] itself, we had set our goals for the future. We feel content to have not only achieved our set goals, but also advanced far beyond.

## Learn

The total number of issues published annually was increased from the previous four to six. The journal is now being published and delivered so regularly that the editorial office receives complaints of non-receipt even in the event of a delay by just 1 week!

When we realized that the inability to pay for relevant articles was a handicap for some aspiring authors, the editorial office stepped in to provide full text of any article to members for research purposes.

Our mantra of “Open Access” became more meaningful by the act of locating, formatting and uploading the full text of all the back issues of the *IJO*. This has resulted in an enhanced visibility of our authors’ efforts from its inaugural issue in 1953 to time immemorial!

**Figure 2:**
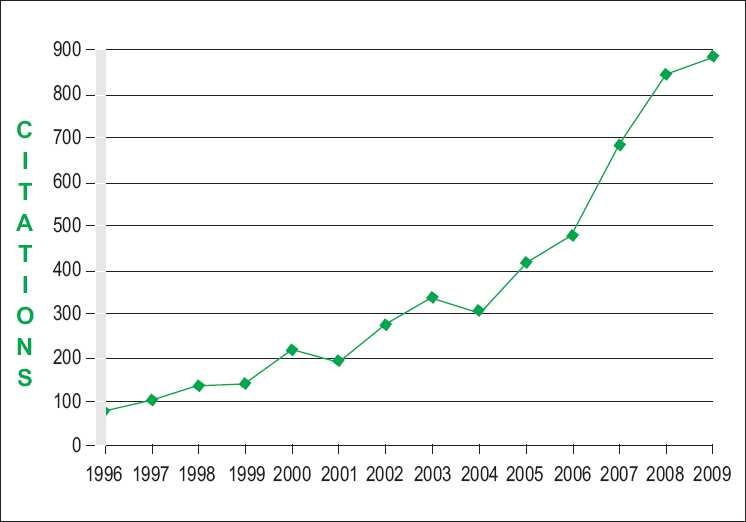
The increasing citations of the Indian Journal of Ophthalmology (Source: SCOPUS)

We added a large measure of ***“Scientific Dignity”*** to our contributors by virtue of indexing the *IJO* by more than 30 indexing systems, the Science Citation Index and PubMed Central being some of the most prestigious of this orderly procedure of scientific classification of all known Ophthalmic Gyan [Fig. 2]. We state, with a sense of due pride, that we expect the ***Impact Factor***, as cited by the Journal Citation Report, to be around 1. This is, by any means, a commendable achievement for a new entrant.

## Educate

In order to fulfill our declared objective of helping our members to conduct quality research and inculcate the art of proper scientific writing, we decided to initially focus upon getting their basics clear. For the first time in its history, ***the IJO crossed the threshold from print to pen***. We launched a novel teaching module – ***the research methodology workshop***. We conducted seven such workshops in the larger metros and, as they gained in popularity with over 150 attendees, today there is an increasing demand to hold them even in smaller cities and towns.

Researchers and authors go hand in hand in improving the quality of any journal. To attract good researchers from India to the *IJO*, we reduced the color processing charges levied to the authors from ‘10,500/- to ‘2000/- (up to six photographs) in the very first year and have not increased the charges ever since.

With an aim to further classify educational data, the editorial team planned and published symposia on *Management of Uveitis, Anti-Vascular Endothelial Growth Factor, Infective Keratitis, Acquired Immuno-Deficiency Syndrome, Manual Small Incision Cataract Surgery*, and *Glaucoma*. CDs on *Infective Keratitis, Glaucoma* and *Research Methodology* were also distributed through IJO as an innovatively handy and far reaching educational tool. The content of the symposia and CDs were very useful and highly appreciated.

We commissioned a series of *focused abstracts* on diverse topics such as, *Uveitis, Intraocular lenses (IOLs), Keratoconus, Keratoplasty procedures and Congenital Cataract*. This has served to bring together a wide body of information from several sources into one single domain, thus furthering the ability of our members to seek relevant data and references.

Along with this issue, we are pleased to provide you, again for the first time, a non-sponsored ***“Supplementary Issue on Glaucoma” that incorporates two Editorials with no less than 22 invited review articles from 18 major institutes across the globe!*** Being the first among several others planned (Community Ophthalmology, Refractive Procedures and Pediatric Ophthalmology are in the pipeline), we are sure that this supplement will have both a historical and collector’s value too.

## Evolve

**Figure 3:**
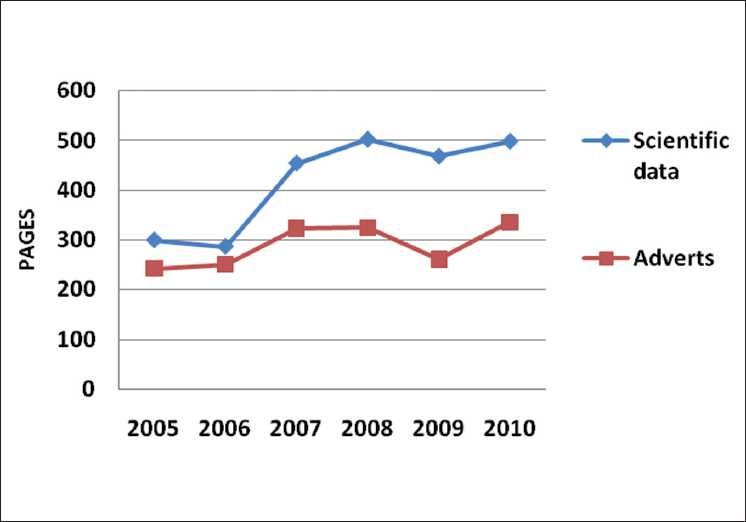
Break up of contents in IJO

No one can deny that the *IJO* has evolved tremendously, and for the better, these past 6 years [Fig. 3].

Our submissions and subsequent procedures are cent percent online. A total of 189 articles were published in the year 2010. This is a record of sorts for the *IJO*.

**Figure 4:**
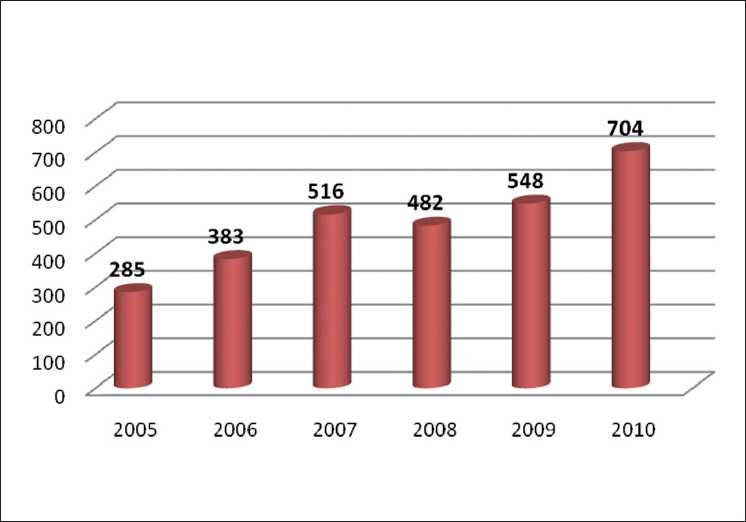
Year wise submission of articles in IJO

Buoyed by a bounty of submissions this year [Fig. 4], we happily raised the quality bar for acceptance of articles still higher. In spite of this increased workload, the performance of the editorial office has shown a steady improvement.

**Figure 5:**
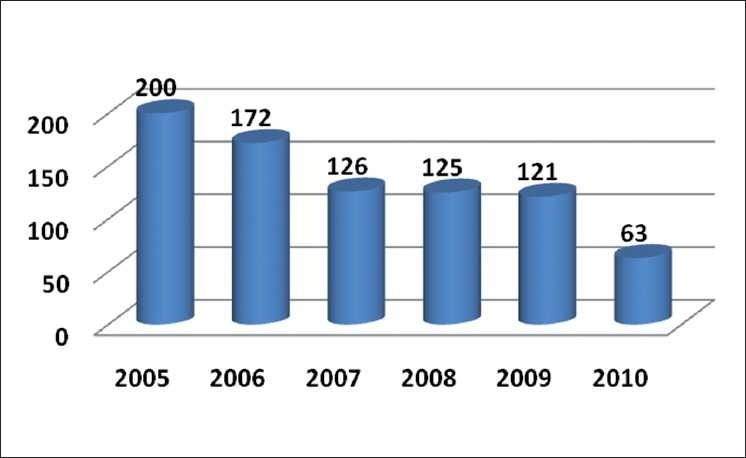
Days from acceptance ot publication

**Figure 6:**
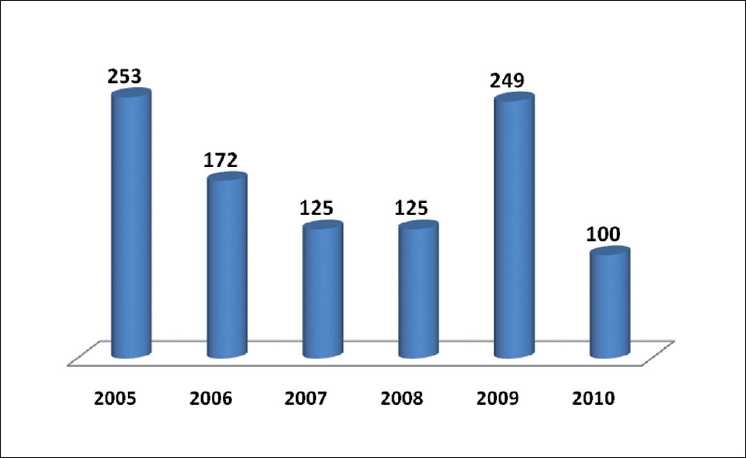
Days from submission to acceptance

The turnaround time from submission to publication has been reduced from over 450 days in 2005 to just 23 weeks by 2010 [Figs. 5 and 6].

**Figure 7:**
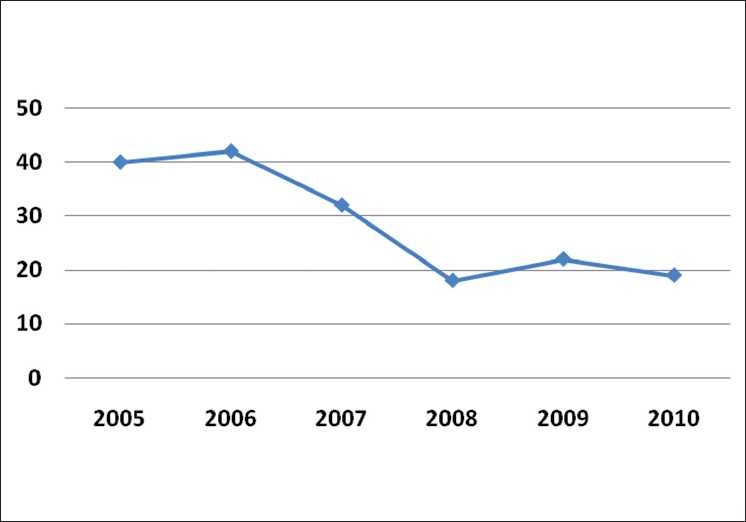
Review time in days

**Figure 8:**
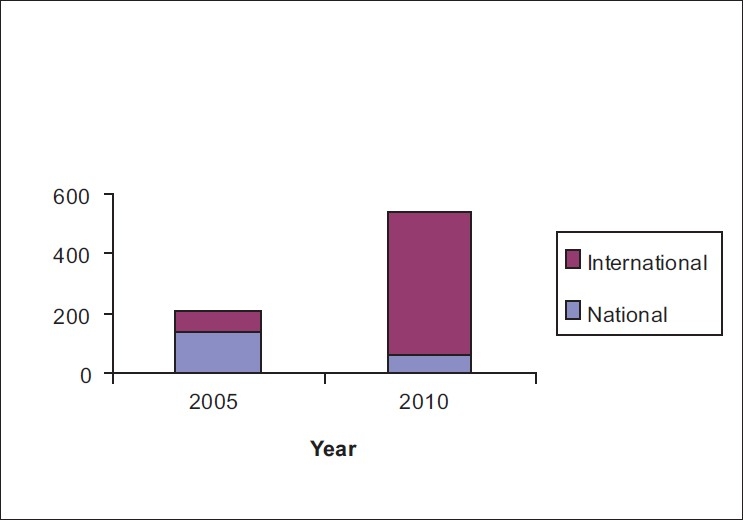
IJO reviewers’ data base

The role of reviewers can never be undermined in elevating the standard of any journal. *IJO* has, over time, acquired a strong support of hundreds of national and international reviewers [Figs. 7 and 8].

**Figure 9:**
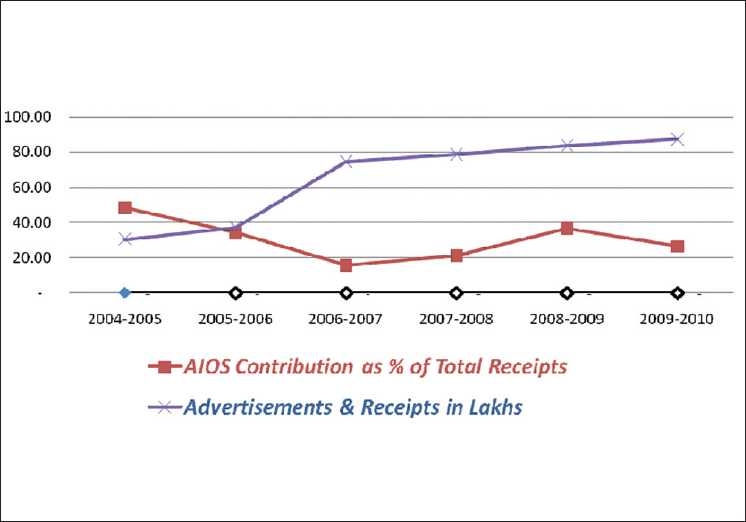
IJO financial highlights

From being on a precarious financial perch, dependent significantly on AIOS funds in 2005 [Fig. 9], the *IJO* has not only willingly reduced its share of the AIOS funds, but also elevated its position to such heights that in the last 6 years, it has increased its surplus funds by more than 2500%.

**Figure 10:**
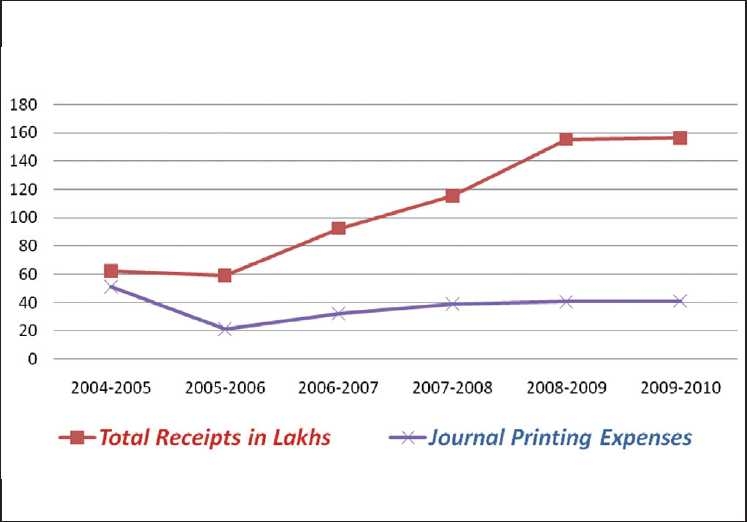
IJO receipts Vs printing expenses

We have constantly endeavored to reduce costs, while improving the quality of output [Fig. 10].

**Figure 11:**
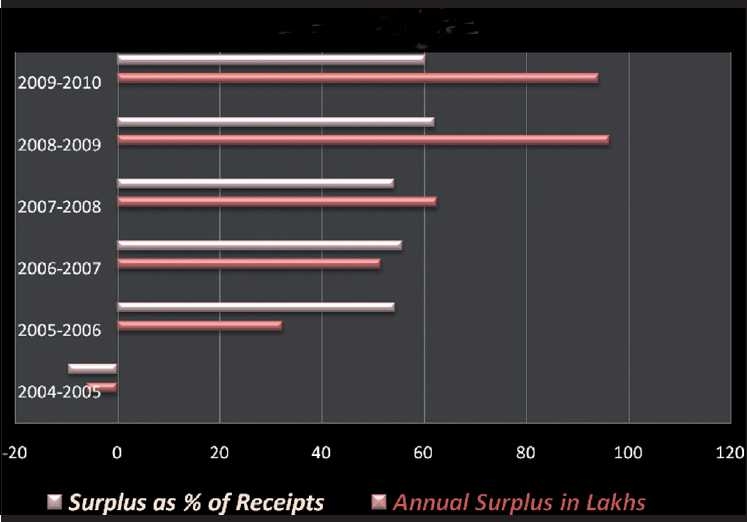
IJO surplus

We inherited a negative surplus. We bequeath a financially sound product, capable of self-sustenance! [Fig. 11] Need we say more?

The *IJO* has, over the past 72 months, carved out a special and unique space on the AIOS firmament.

**Figure 12:**
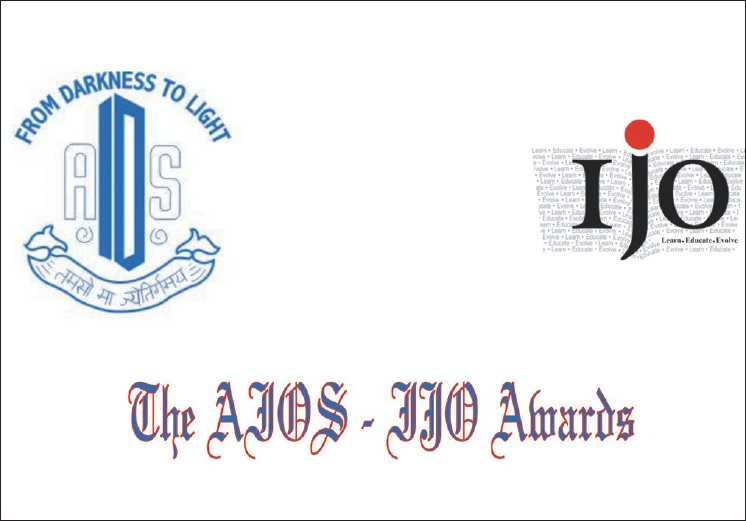
The AIOS - IJO awards

We instituted ***The AIOS-IJO Awards*** in the year 2009, thus taking one more step toward appreciating our authors and providing a permanent recognition of their work [Fig. 12]. We are sure that these awards, carrying both a cash prize and a citation certificate, will act as a further incentive for more and more authors to make *IJO* their first choice for publication.

We are sure one and all will appreciate the innovative features on our website, particularly of the ***IJO “E BOOK”***, as we prefer to call it. It raises screen-based reading to a new level. Be that as it may, we note with dismay that computer penetration amongst our members stands at a woeful 44%.

Thankfully or otherwise, the printed word is still with us and promises to stay for a long time to come.

The print format of the *IJO* is unlikely to face redundancy in the near future, thus reinforcing the belief that our conditioned learning reflexes still need a printed or written format.

## Future

The present editorial team, as the co-runner in the success story of *IJO*, would like to see *IJO* being published monthly with its “Impact Factor” amongst the top 10 Ophthalmology journals in the world. Many enthusiasts may label this target as a very modest expectation from the next team, but we believe in taking one step at a time so that we do not lose balance before taking the next leap.

## Conclusion

As we inevitably hand over charge to the next team, we look back with a sense of gratitude to all our stakeholders – contributors, reviewers, editorial assistants, editorial committee members, editorial board members, AIOS office bearers, managing editor, publisher, printer, and above all, you – the readers. Thank you one and all for scripting our success story so well, so agreeably.

This is truly the saddest of times that the voyage has to end.

It is also the best and most opportune time to hand over the baton.

We look back at this wonderful journey with a sense of fulfillment and a dash of justifiable (and therefore, forgivable) pride, secure in the knowledge that our mantra will outlive us.

**Our Mantra is work, hardwork, teamwork, pleasure in work.**
